# Radiation-induced kidney toxicity: molecular and cellular pathogenesis

**DOI:** 10.1186/s13014-021-01764-y

**Published:** 2021-02-25

**Authors:** Richard Klaus, Maximilian Niyazi, Bärbel Lange-Sperandio

**Affiliations:** 1 Division of Pediatric Nephrology, Department of Pediatrics, Dr. v. Hauner Children’s Hospital, University Hospital, LMU Munich, Lindwurmstr. 4, 80337 Munich, Germany; 2 Department of Radiation Oncology, University Hospital, LMU Munich, Munich, Germany; 3 German Cancer Consortium (DKTK), Partner Site Munich, Munich, Germany

**Keywords:** Radiation nephropathy, Total body irradiation, Radiotherapy, Renal fibrosis, Cellular senescence, DNA damage, Renal inflammation

## Abstract

Radiation nephropathy (RN) is a kidney injury induced by ionizing radiation. In a clinical setting, ionizing radiation is used in radiotherapy (RT). The use and the intensity of radiation therapy is limited by normal-tissue damage including kidney toxicity. Different thresholds for kidney toxicity exist for different entities of RT. Histopathologic features of RN include vascular, glomerular and tubulointerstitial damage. The different molecular and cellular pathomechanisms involved in RN are not fully understood. Ionizing radiation causes double-stranded breaks in the DNA, followed by cell death including apoptosis and necrosis of renal endothelial, tubular and glomerular cells. Especially in the latent phase of RN oxidative stress and inflammation have been proposed as putative pathomechanisms, but so far no clear evidence was found. Cellular senescence, activation of the renin–angiotensin–aldosterone-system and vascular dysfunction might contribute to RN, but only limited data is available. Several signalling pathways have been identified in animal models of RN and different approaches to mitigate RN have been investigated. Drugs that attenuate cell death and inflammation or reduce oxidative stress and renal fibrosis were tested. Renin–angiotensin–aldosterone-system blockade, anti-apoptotic drugs, statins, and antioxidants have been shown to reduce the severity of RN. These results provide a rationale for the development of new strategies to prevent or reduce radiation-induced kidney toxicity.

## Radiation therapy

Radiation therapy (RT) alone or in combination with chemotherapy, surgery or both is a major treatment option for solid malignancies. Globally, the incidence of cancer rises because human lifespan and exposure to cancer risk factors increase [1]. In 2018, approximately 18 million people were diagnosed with cancer and more than 9 million people died of cancer [2]. Predictions conclude that cancer may become the leading cause of death in the twenty-first century, and more than 60% of cancer patients will receive RT throughout their therapy regimen [3]. RT can be administered as external beam radiotherapy (teletherapy). Internal radiation therapy includes brachytherapy, where a source of radiation is implanted [4], intraoperative radiotherapy, or radionuclide therapy, where therapeutic radionuclides are administered parenterally [5].

External beam RT using linear accelerators is the most common form of RT [6, 7]. With the technical advances of recent years, novel radiation techniques have been introduced such as intensity modulated radiotherapy (IMRT) and its rotational subform volumetric modulated arc therapy (VMAT) [8, 9]. In parallel, image-guided radiotherapy (IGRT) has been improved and most commonly used options are kV/MV planar imaging, cone beam CT, ultrasound, surface scanners with X-ray imaging, and magnetic resonance guided RT (MRgRT) [10–12]. And yet, particle therapy with proton or heavy ion beams can be used in specific indications as their depth dose profile allows for steep gradients beyond the target. This process has been accompanied by improved target volume delineation, inverse radiation treatment planning, and the option of adaptive radiation dose delivery [13, 14]. Apart from targeted RT, total body irradiation (TBI) is a widely used technology. It is one of the conditioning regimens to prepare patients for bone marrow transplantation (BMT). Annually there are more than 20,000 BMT worldwide [15].

## Radiation toxicity

First, general principles of radiation toxicity are presented. Specific evidence for the contribution of those putative pathomechanisms in radiation nephropathy will be discussed separately later.

Although radiation therapy is very effective to control tumour growth and prolong overall survival, it has adverse effects on healthy tissue within the field of radiation. Selectively reaching only the cancerous tissue remains one of the major challenges in RT [16]. Toxicity to healthy tissue limits the applied doses of RT and thus leads to suboptimal tumour control. Furthermore, the combination with chemotherapy increases normal-tissue toxicity and therefore leads to even further reduction of the tolerable maximum dose [7].

The main target of radiation therapy is the DNA. Ionizing radiation causes direct damage by destroying chemical bonds and knocking out electrons. Ionizing radiation also causes indirect damage through generation of reactive oxygen species (ROS) [17]. When ROS outweigh antioxidants, oxidative stress arises. ROS injure cellular macromolecules e.g. lipids, proteins or DNA [17, 18]. DNA double-strand breaks (DSB) are the most severe event of RT [19]. Cells sense DSB by a system called DNA damage response (DDR). The DDR is initiated within minutes after irradiation and activates cell cycle checkpoints and DNA repair in order to achieve survival. When reparation processes are unsuccessful, DSB finally cause genomic instability, cell death, or cellular senescence [20]. The reaction to DSB depends on the affected tissue and the integration of DDR in the affected cells. In rapidly reproducing tissues (such as the targeted tumour cells, but also hematopoietic cells and mucosal epithelium) unrepaired DSB and subsequent rounds of aberrant mitosis culminate in a morphotype of mitotic catastrophe and cell death [21]. This reflects the therapeutic benefit with killing of tumour cells, but also the acute clinical toxicity of radiotherapy as a consequence of normal tissue cell death. This usually takes place within the first two weeks. Slowly reproducing tissues (such as fibroblasts) rather react with prolonged cell cycle arrest instead of cell death induction [22]. While acute radiation toxicity is marked by acute cell death, the processes in chronic toxicity are generally characterized by fibrogenesis and extracellular matrix deposition. Those processes are most likely secondary through chronic inflammation and cellular senescence [23]. Inflammation is present for example in gastrointestinal [24] and lung [25] radiation injuries. Fibrotic reorganisation leads to degeneration and decline of the specific organ function [26].

## Functions of the kidney

The kidneys are essential organs to regulate the organism’s fluids, electrolytes, and acid–base metabolism. Kidneys excrete waste metabolites, modulate the blood pressure, produce erythropoietin to stimulate erythropoiesis, and activate vitamin D [27]. Following radiation injury, kidney dysfunction leads to hypertension, anaemia, and osteodystrophy. Toxic waste metabolites accumulate and cause uraemia, electrolyte disorders such as hyperkalaemia, hyperphosphatemia, hypocalcaemia and ultimately chronic renal failure [28]. End stage renal disease (ESRD) with loss of kidney function results in renal replacement therapy with the need for dialysis or renal transplantation [29].

## Clinical course of radiation nephropathy

The clinical course of radiation nephropathy was first described by Luxton et al. [30] (Table 1). Besides very high radiation doses above 50 Gy, which are irrelevant to human experience, there are no symptoms or clinical signs in the first 6 months after irradiation. This is the so called latent period of RN. First clinical damage becomes apparent in the acute phase [6–18 months] after irradiation. Luxton et al. observed first clinical signs 6 to 13 (mean 8.5) months after irradiation [30]. Chronic kidney damage becomes clinically apparent more than 18 months after RT.

**Table 1:** Clinical stages of radiation nephropathy

Type	Time after radiotherapy	Symptoms
Latent period	< 6 months	No symptoms or clinical abnormalities
Acute radiation nephropathy	6–18 months	Signs of glomerular pathology Oedema, azotaemia, proteinuria, hypertension, hypertensive crisis, fatigue, anaemia
Chronic radiation nephropathy	> 18 months	Signs of chronic kidney disease Hypertension, albuminuria, anaemia, chronic renal failure, small atrophic kidneys

Acute RN may start symptomless. Azotaemia or proteinuria might be detectable before symptoms occur. When symptomatic, patients may present with fatigue, oedema, headaches and severe anaemia disproportionate to impairment of kidney function [31]. Even hypertensive crisis with encephalopathy or congestive heart failure may appear [31]. Chronic RN (CRN) presents with hypertension, proteinuria and chronic renal failure [32]. Kidney atrophy, that is typical in CRN can be measured by kidney volume loss [33] (Table 1). In conclusion, CRN is clinically indistinguishable from chronic kidney disease (CKD) of any other cause. It could be argued that the decline in kidney function after RT in patients with malignancies is a consequence of chemotherapy or multiple nephrotoxic drugs such as antimicrobials. However, there are two large series of patients with BMT without TBI, where no chronic kidney disease was reported [34, 35]. In addition, CRN is less frequent in patients undergoing TBI with partial kidney shielding [36]. Observations of patients with RT from the 1970s have shown that the latency for CRN can be as long as 8–19 years [37]. When patients with CRN reach ESRD, the survival is much worse than for other causes of ESRD [38].

## Dose thresholds and special implications for kidney radiation toxicity

The severity of clinical features of RN depends on the kind of application (e.g. partial vs. total body irradiation, internal beam vs. external beam radiation), the applied dose and the affected kidney volume [39, 40]. Clinically apparent kidney injury promptly after irradiation is observed only at higher doses than currently used. It has been reported in animal models using > 50 Gy [41, 42]. The doses used in clinical settings in total or partial body irradiation and internal radiation are much lower—as specified below—and cause late effects involving the glomeruli, tubulointerstitium and renal vasculature [43]. There are no data for specific thresholds of radiation therapy for the different compartments of the kidney.

In partial body irradiation, clinical studies recommend to keep the mean dose for both kidneys below 18 Gy to limit renal toxicity. 15 to 17 Gy in 2 Gy fractions were considered safe, doses of 23 Gy can cause CKD in 5% of cases, and 28 Gy cause CKD in 50% of cases [44]. In a study with 19 patients with irradiated paraaortic lymph nodes in gynaecologic tumors, those with a mean dose for both kidneys of 18 Gy stayed clinically asymptomatic for 12 to > 48 months of observation time [45]. There are data from the Quantitative Analyses of Normal Tissue Effects in the Clinic (QUANTEC). QUANTEC data show that up to 50% of patients develop clinically relevant kidney dysfunction if both kidneys are irradiated with a mean dose > 18 Gy. If less than 20% of the kidney volume are exposed to 28 Gy (V28 < 20%) only < 5% of patients will develop a clinically relevant kidney dysfunction [40]. Recently, in a series with 663 patients published in 2020 only 2% of patients with adjuvant radiotherapy for gastric cancer developed renal function impairment. The volume of the kidney receiving a dose of 20 Gy (V20) is predictive for renal function impairment [46].

For patients undergoing TBI in preparation for BMT, radiation nephropathy with CKD and arterial hypertension still belongs to the common late sequelae. However, due to newer workflows and techniques numbers have decreased [31, 47, 48]. In a series from 1993 up to 25% of patients developed radiation nephropathy [31]. In a meta-analysis in 2006 Kal et al. suggested the biologically effective dose (BED) should be less than 16 Gy in fractions < 2 Gy [49]. Below this threshold, the clinical renal dysfunction is close to zero. Above a BED of 21 Gy the frequency of clinical renal dysfunction exceeds 20%. Therefore, the authors suggest kidney shielding above a BED of 16 Gy [49].

Fractionation of doses allows higher total dosages, as it allows repair of sublethal damage, repopulation, reoxygenation and reassortement of cells in the cell cycle. In current standards external radiation is applied in fractions of 1.5–2 Gy [16]. Estimates suggest, that in humans an exposure to a single dose of 4 Gy may lead to renal injury [50]. Exposure to high-dose-rate single doses are no longer used in clinical practice, but might occur in radiation accidents or radiological terrorism. Anno et al. estimated that a 7 Gy single dose of high-dose-rate ionizing radiation can be survived under currently available optimal medical support [51]. Decades ago the 50% lethal doses in Nagasaki and Chernobyl were 2–4 Gy and 5–6 Gy respectively. These findings imply that radiation nephropathy would be a relevant issue in survivors of such scenarios [52].

In radionuclide therapy—a form of internal RT used for various cancers including neuroendocrine tumors—the kidneys are especially susceptible for radiation toxicity because of the glomerular filtration, tubular absorption and retention of the proximal tubules of radionuclides [5]. After glomerular filtration, approximately 3% of the total activity is reabsorbed and retained by the proximal tubuli, leading to prolonged radiation exposure of the kidneys [53]. Severe nephrotoxicity with up to 14% grade 4–5 adverse events (ESRD or death) occurred in patients treated with radionuclides [50]. Due to the different nature of the application dose thresholds differ from external radiation therapy. While external radiation is homogeneously applied at high-dose-rates, radionuclides are heterogeneously distributed through the organ and dose rates are much lower, variable with time and with exponential decrease. For ^90^Y-DOTATOC and ^177^Lu-DOTATATE doses less than 40 Gy were safe for patients without any risk factors, while for patients with risk factors for CKD—mainly hypertension and diabetes—a threshold of 28 Gy was recommended [54]. To reduce proximal tubular absorption co-infusion of positively charged amino-acids such as l-lysine and l-arginine is recommended. It reduces the absorbed dose ranging from 9 to 53%. Tailoring dosimetry to individual patients is an important measure to improve therapeutic potential and reduce renal toxicity.

Although dose thresholds and BED of the kidney may differ among the different modes of radiation therapy, it is not to be expected that the molecular and cellular pathomechanisms are different. The injuring stimulus—ionizing radiation—remains always the same. Hence, the pathomechanisms are discussed regardless of the application form.

## Histopathology

The acute morphologic changes in the kidney after RT are mainly vascular and glomerular. Loss of endothelial cells with subendothelial expansion is an early sign of irradiation injury. Capillary loops are occluded and congested. Both thrombosis and casts of degenerated erythrocytes are present in the glomerular capillaries. Mesangiolysis is another common finding. Electron microscopy shows endothelial cell injury and subendothelial widening of the glomerular basement membrane [55, 56]. Chronic changes are characterized by an increase in renal interstitial fibrosis and a loss of nephron mass. Sclerosis of interlobular and arcuate arteries, tubular atrophy and glomerular scarring are late features of RN [56, 57].

## Pathomechanisms of radiation toxicity

The molecular and cellular pathomechanisms of chronic kidney disease (CKD) in general are of high interest, because CKD affects more than 700 million patients worldwide [58]. While many aetiologies for CKD exist, there seems to be a common final pathway with glomerulosclerosis, renal interstitial fibrosis, and tubular atrophy with impairment of kidney function [23]. Chronic inflammation and cellular senescence are driving factors for fibrotic processes in almost all aetiologies of CKD [59, 60].

**Fig. 1:**
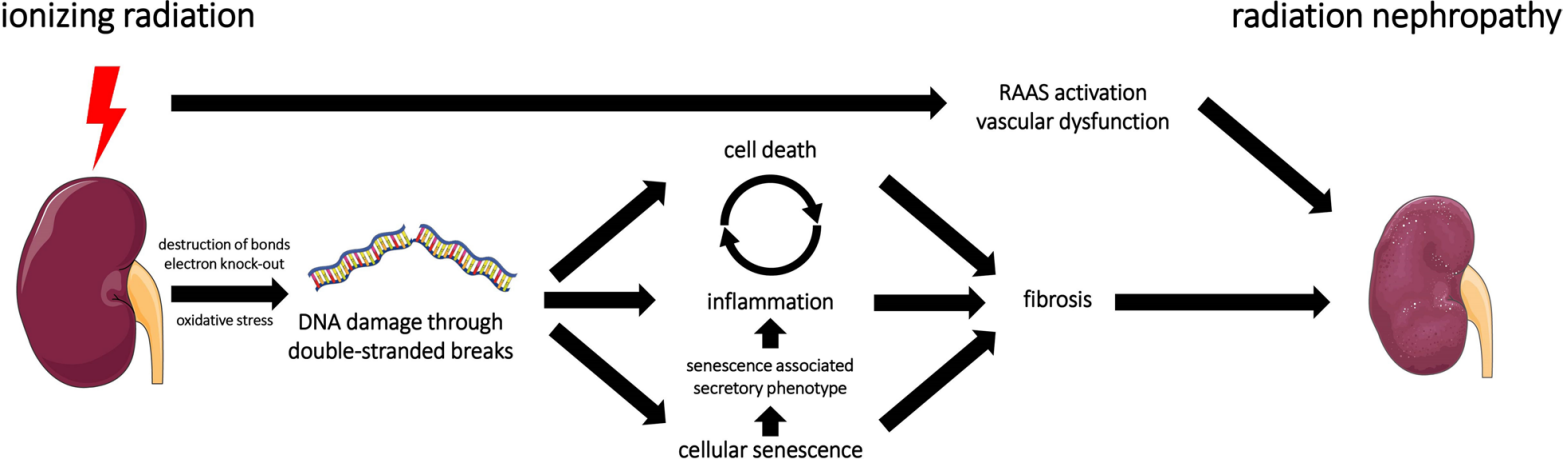
Interaction of putative pathomechanisms in radiation nephropathy. Ionizing radiation induces DNA double-strand breaks (DSB) as well as renin–angiotensin–aldosterone system (RAAS) activation and vascular dysfunction. RAAS-activation and vascular dysfunction contribute to radiation nephropathy. DSB are induced directly through destruction of bonds and knock-out electrons and indirectly through oxidative stress. DSB trigger acute cell death, inflammation, and cellular senescence. Acute cell death and inflammation may enter a viscous cycle, although data on chronic inflammation in radiation nephropathy is conflicting. Cellular senescence promotes inflammation via the senescence associated secretory phenotype (SASP), which consists of pro-inflammatory cytokines. Cell death, possibly inflammation, and cellular senescence lead to loss of nephron mass and interstitial fibrosis, the hallmark of radiation nephropathy

In RN, the initial renal cell injury, which may start the cascade towards CKD, are DSB of the DNA through ionizing radiation, either by direct ionization events in the DNA or indirectly via mediation of water ionization products and/or reactive oxygen species [61] (Fig. 1). This acute DNA damage can cause immediate cell death in the kidney [62–64]. In cancer patients undergoing RT, transcriptome profiling studies show nephrotoxicity with upregulation of genes for renal necrosis and apoptosis [65]. In cells surviving the acute phase, DNA repair mechanisms are highly activated [66]. Even when cells do not die from acute damage, misrepaired DSB can still induce cell death or cellular senescence in the long term [20]. Cytokines released upon cell death [67], cellular senescence [60], and ionizing radiation itself [7] trigger chronic inflammation. Finally, chronic inflammation and cellular senescence may lead to renal fibrosis [23].

Thus, the putative molecular and cellular signalling pathways of RN start within the spectrum of DNA damage and its repair mechanisms in the kidney. Cell death, oxidative stress, vascular dysfunction, cellular senescence, inflammation, release of profibrotic agents, and Renin–angiotensin–aldosterone system (RAAS)-activation are the putative pathomechanisms in radiation nephropathy (Table 2). Those pathomechanisms and the currently available data will be discussed in the following paragraphs.

**Table 2:** Putative pathomechanisms and therapeutic agents in radiation nephropathy

Putative pathomechanisms	Putative therapeutic agents	Species	Sources
Oxidative Stress	N-Acetylcystein Atorvastatin Montelukast	Mouse, rat	Supporting evidence: [68–71] Opposing evidence: [72–75]
Renin–angiotensin–aldosterone system (RAAS)	ACE-inhibitors Aldosterone-antagonists	Human, mouse, rat	[76–82]
Cellular senescence (CS)		Rat	[83]
Inflammation	Montelukast	Mouse, tibet mini pigs, rabbits, primates	supporting evidence: [69, 84, 85] opposing evidence: [56, 57, 86, 87]
Fibrosis	ACE-inhibitors Epoxyeicosatrienoic acids	Human Mouse, primates	[76] [56, 69]
Vascular dysfunction	Epoxyeicosatrienoic acids	Human, mouse, rat	[88–91]

## Oxidative stress

Oxidative stress (OS) is present when reactive oxygen species (ROS) outweigh enzymatic and non-enzymatic antioxidants. ROS react with lipids, proteins, and DNA [17]. This leads to cellular damage and senescence [92]. In radiation-induced tissue damage, the role of OS in the acute phase with DNA-damage is well-established [17]. Furthermore, OS is an important pathomechanism in CKD, CKD-complications and CKD-progression [93, 94]. Hence, OS might also play a role in RN.

However, it is important to understand the difference between OS generated promptly after the irradiation and OS in the latent period of RN. In order to prove, that OS plays a role in the aetiology of chronic radiation nephropathy, OS needs to be present in the latent period. Such data are rare and still conflicting. Zhao et al. hypothesised that chronic OS is responsible for RN. The authors showed upregulated DNA oxidation in viable glomeruli and tubuli in rats 4 to 24 weeks after single dose irradiation with 20 Gy [71]. By contrast, Lenarczyk et al. found no evidence for chronic OS in the latent period in rats that underwent TBI with either 18.8 Gy in 6 fractions or 10 Gy in a single dose. There was no evidence for lipid peroxidation or protein oxidation in the urine in the first 42 days. After 89 days renal tissue did not show evidence of DNA or protein oxidation [74]. Likewise gene expression analysis revealed no relevant increase in genes related to OS in the first 49 days after single-dose TBI with 10 Gy [73]. Rats became symptomatic with proteinuria approximately 6 weeks after irradiation and uremic morbidity occurred after 26 weeks. Hence, the investigated time points were in the latent phase of experimental RN, showing no evidence for OS in the latent period.

Cohen et al. found no mitigation of RN for the anti-oxidative agents deferiprone, genistein and apocynin, when administered in the latent period after 10 Gy single-dose TBI in rats [72]. By contrast, protection against OS was shown in experimental settings, when the anti-oxidative substances were administered before irradiation. Mercatepe et al. showed that the ROS-scavenging anti-oxidant N-acetylcystein increased glutathione-levels in 6 Gy TBI irradiated rats. N-acetylcystein mitigated histopathological RN and reduced caspase-3 expression. In this study N-acetylcystein was administered 5 days before until 2 days after TBI. The authors concluded that the benefits were obtained by reducing OS [68]. Similar results came from Amiri et al. using lipid-lowering statins in experimental RN. Beside effects on lipid metabolism, statins have anti-inflammatory, anti-apoptotic and antioxidant effects [95]. Daily treatment with atorvastatin 7 days before 2 Gy TBI decreased lipid peroxidation as a marker of OS, improved kidney function, reduced caspase 3-expression, and ameliorated tubular damage in mice [70]. Nephroprotective and antioxidant effects were also shown for leukotriene receptor antagonists. Montelukast is a selective leukotriene CysLT1-receptor antagonist, that was developed for respiratory dysfunction. This anti-inflammatory drug also ameliorated OS [96]. Hormati et al. found that montelukast administered 2 weeks before 3 Gy TBI in mice was able to reduce OS and mitigate RN [69].

Altogether, the data on OS in radiation nephropathy show clear evidence, that OS plays an important role in damaging the DNA promptly after irradiation. In addition, the application of anti-oxidants before irradiation consequently mitigates RN. By contrast, in the latent phase OS was neither detectable in a significant amount, nor could pharmacological attenuation of OS mitigate RN. Therefore, OS does not seem to play a role after the very early moment of irradiation. However, the consequences of OS—DNA damage, cell death, and induction of cellular senescence—are crucial in RN.

## Renin–angiotensin–aldosterone system

The renin–angiotensin–aldosterone system (RAAS) consists of enzymes and their peptide substrates and involves multiple organs. RAAS is an important regulator of the blood pressure and electrolytes. Renin is produced in the kidneys. It cleaves angiotensinogen from the liver into angiotensin I (AT I), which is then converted to angiotensin II (AT II) by the angiotensin-converting enzyme (ACE), which originates from the lungs. However, all individual RAAS-components are present within the kidneys and are called intrarenal RAAS. Intrarenal activation of RAAS plays a critical role in renal diseases, especially in renal hypertension [97].

RAAS-inhibition with captopril mitigated RN in a randomized controlled trial in patients after 14 Gy TBI in 9 fractions with kidney shielding yielding a total dose of 9.8 Gy in preparation for BMT. Captopril or placebo were administered after host engraftment and improved 1 year-GFR and overall patient survival [76]. RAAS-inhibition was beneficial in animal models of RN (single-dose 10 Gy TBI in rats, ^177^Lu-DOTATATE application in mice) [77–79]. The mitigation by RAAS-inhibition was more effective in animal experiments than in patients after BMT. The most likely explanation is, that in patients TBI is not the only nephrotoxic agent in the process of BMT. Chemotherapy, infection and anti-infective drugs contribute to CKD after BMT and RAAS-inhibition does not mitigate all forms of renal injury.

RAAS-inhibition is also beneficial in radiation injuries in lungs [98] and brain [99]. These findings urgently raise the question, whether RAAS plays a mechanistic role in RN after RT. So far, no solid evidence for RAAS-induction in RN exists. Cohen et al. showed no RAAS-activation at all with normal renin activity, normal renin protein levels, and normal values for serum and intrarenal AT II after 17 Gy TBI in 6 fractions [82]. Renal cell membrane AT II receptor binding was equally seen in rats after TBI with 18.8 Gy or 20.5 Gy given in six fractions over 3 days and the control group [81]. Aldosterone is a peripheral component of the RAAS-system involved in various types of renal injuries [100]. In one study no elevation in aldosterone was shown after 10 Gy single-dose TBI in rats and the aldosterone-antagonist spironolactone did not mitigate RN [79]. However, another group found spironolactone to mitigate RN after internal alpha-particle irradiation in mice [101]. It seems, that just like AT II-blockade, aldosterone-antagonists may mitigate RN, although aldosterone itself is not upregulated.

The clearly beneficial effect of RAAS-inhibition with no measurable increased RAAS-activity suggests that either normal RAAS-activity is harmful in irradiated subjects or RAAS-opposing systems such as nitric oxide (NO) decrease after irradiation [79]. There is evidence of NO-reduction in RN in rats after 17 Gy TBI in 6 fractions over 3 days, and RN could be mitigated by captopril [102]. In general, RAAS-inhibition stabilizes progress of numerous kidney diseases of different aetiologies. Its nephroprotective effect is mediated by the reduction of intraglomerular pressure and hence reduced proteinuria with consecutively less tubulointerstitial damage [103]. In conclusion, RAAS-inhibition might have a protective effect by reducing intraglomerular pressure, renal fibrosis, and balancing the NO-reduction in RN. RAAS blockade is therefore a very promising strategy for RN therapy.

## Cellular senescence

Cellular senescence (CS) is the combination of cell cycle arrest, suppression of apoptotic pathways, a high metabolic activity and a senescence-associated secretory phenotype (SASP). SASP includes an increased secretion of IL-1, IL-6, IL-8, connective tissue growth factor, transforming growth factor, vascular endothelial growth factor and TNF-α [104, 105]. While it is part of the normal chronological aging process, characterized by the attrition of telomeres, premature senescence is induced by stress factors, such as ionizing radiation directly or indirectly by OS [106, 107]. In the brain [108], heart [109] and lungs [98] CS contributes to radiation-induced organ damage. In CKD of other aetiologies than CRN, CS is also a suggested pathomechanism [60]. In experimental RN with single-dose 18 Gy in rats, CS has been shown in glomerular endothelial cells and in podocytes of rats, underlining the impact of CS in RN. Glomerular endothelial injury was dominant, resulting in an increase in thrombotic microangiopathy, collapsing glomeruli, and a decreased number of endothelial cells in experimental RN. The renal cells demonstrated upregulated markers of cellular senescence (p53, p21, p16), cell cycle arrest, and had a SASP with increased IL-6 secretion. TNF-α, IL-8, and VEGF-A secretion were not significantly increased. Glomerular damage and impairment of kidney function were found in this experimental model of RN [83]. Thus, cellular senescence seems to be activated in RN.

## Inflammation

Inflammation has been proposed as a mechanism for RN because it is present in other radiation injuries such as gastrointestinal radiation injury [110] and radiation pneumonitis [25]. Furthermore, mechanistically inflammation links renal cell injury and CKD. Necrotic tubular cells release damage-associated molecular patterns (DAMPs) and trigger secretion of pro-inflammatory cytokines and chemokines in tissue-resident cells and recruited leukocytes [111]. Macrophages for example produce cytokines such as TNF-α and IL-6. These inflammatory responses lead to even more cell death and fuel a viscous cycle of cell death and inflammation [67], followed by a decline in kidney function and the initiation of renal fibrosis [112]. However, there is only little data on active inflammation in RN. Pro-inflammatory cytokines, such as TNF-α, IL-1β and interferon-γ were found to be the primary upstream regulators of the upregulated transcripts in mice after ^177^Lu-Octreotate-admission [84]. TNF-α expression levels increased and correlated with the metabolic activity detected in [^18^F]-FDG-PET-CT in tibet mini pigs after 2, 5, 8, 11 and 14 Gy single-dose TBI [85]. Further evidence for the involvement of inflammation in RN derives from the observation that montelukast was able to mitigate RN in mice after 3 Gy single-dose TBI in a dose-dependent way [69]. Montelukast has anti-inflammatory effects via inhibition of nuclear factor-κB activation and reduction of anti-inflammatory cytokines, such as TNF-α and IL-6 [113].

By contrast, there are recent studies performed in rhesus macaques, demonstrating that inflammation plays only a minor or no role in RN. Van Kleef et al. investigated chronic RN in macaques by histological analysis 6 to 8 years after single-dose irradiation of 4.5–8.5 Gy or two fractions of 5 Gy. In comparison to age-matched controls there was no significant difference in leucocyte infiltration in the kidney and only slightly increased numbers of macrophages were present in the renal cortex [57]. However, after such a long time period, it is to be expected, that inflammation has already stopped and fibrosis has taken over. Parker et al. exposed macaques to partial-body irradiation at 10, 11, or 12 Gy with 5% bone marrow protection. Inflammatory cell infiltration or increased macrophage populations were no prominent histological features approximately 100 days after irradiation [56]. Very recently, Cohen et al. showed no relevant cellular inflammation in the kidneys 180 days after 10 Gy partial body irradiation in macaques with either 5% bone marrow sparing or 2.5% bone marrow sparing. Cellular inflammation almost never exceeded 1% of the renal parenchymal area [87]. Stimulation of hematopoietic lineages with granulocyte stimulating factor did not change leukocyte infiltration and had neither adverse nor beneficial effects in experimental RN. When tested in irradiated rabbits the potent anti-inflammatory agent prednisolone adversely affected 6 weeks and 9 months survival [86]. Even though it would be possible that non-cellular but cytokine-mediated inflammation plays a role in RN, it seems unlikely, since cells that execute the cytokine-mediated inflammation were not present. To completely dismiss the role of macrophages in RN functional analysis of these populations are currently missing, since tissue-resident macrophages are known to contribute to kidney disease [114].

In summary, current data suggests that inflammation—unlike in other organs—is no significant pathomechanism for RN and the search for treatment options should concentrate on other pathways.

## Vascular dysfunction

Endothelial dysfunction and altered haemodynamics are known features of radiation-induced kidney toxicity [88]. Epoxyeicosatrienoic acids (EETs) are produced in the endothelium by CYP epoxygenase enzymes. EETs derive from arachidonic acid and have been shown to protect kidneys in various models of renal pathologies [89, 90]. Experimental irradiation leads to a decrease of renal CYP epoxygenases and urinary EET levels, to endothelial and vascular damage with afferent arteriolar dysfunction and impaired autoregulatory responses in the kidney. EET-analogues were administered daily from day 2 up to 12 weeks in rats following single-dose 11 Gy TBI. Afferent arteriolar function was improved, hypertension mitigated and renal apoptosis reduced via the Fas/FasL pathway [91]. Thus, EET-analogues seem to mitigate RN by mechanisms other than OS and inflammation and therefore may be promising for future therapies.

## Fibrosis

Renal fibrosis is the formation of scars in the parenchyma. It is a pathologic way of normal wound healing with myofibroblast activation and migration, extracellular matrix deposition and kidney remodelling. Fibrosis is the common final pathway for almost all aetiologies of CKD. The mechanisms leading to fibrosis might be helpful for tissue repair in acute injury, however, when happening persistently in CKD they lead to afunctional tissue und cause a decline in kidney function [23]. Myofibroblasts are the major source of extracellular matrix. Collagen I is the most common matrix protein in renal fibrosis, but types II, IV, V and XV are also found [115]. Transforming growth factor beta (TGF-β) stimulates myofibroblast differentiation in renal fibrosis [116]. Other important stimuli for myofibroblast activation and renal fibrosis are the innate and the adaptive immune system [112]. There is only little histologic and mechanistic data on fibrosis in chronic RN. However, the available data support the hypothesis that fibrosis is present in RN. In macaques renal fibrosis was shown approximately 100 days after 10, 11, or 12 Gy TBI with 5% bone marrow protection. All findings occurred globally throughout the kidney including the cortex and medulla. At time of the necropsy TGF-β was equally increased in irradiated kidneys and controls, which was surprising, given the extensive fibrosis following RT. Those findings suggest an TGF-β-independent effect on renal fibrosis. Renal fibrosis was mostly present in the longest-surviving animals, suggesting it to be a long-term effect, as it is known in other forms of renal injury [56]. RAAS blockade using ACE inhibitors as well as EET-analogues reduced extracellular matrix deposition and renal fibrosis and proved beneficial in experimental RN.

## Conclusion

Although data on the molecular and cellular pathomechanisms in radiation-induced kidney toxicity exist, they are rare. To date, the precise signalling and pathomechanisms are not fully understood. There is some patient data, but most of it comes from experimental models (mostly rats and non-human primates). Ionizing radiation application schemes and doses vary, which further complicate comparability. In many aspects, RN has common features with acute kidney injury transforming into chronic kidney disease. The acute stimulus in RN is ionizing radiation, and the common final stage is renal fibrosis with organ atrophy and decline of kidney function. Oxidative stress and inflammation were proposed to be relevant pathomechanisms in the latent phase, but no solid evidence is present for either of them.

The renin–angiotensin–aldosterone system seems to be a promising candidate. RAAS-inhibitors mitigate the progression of RN in patients and in experimental models. Renal vascular dysfunction is present in RN and can be attenuated by epoxyeicosatrienoic acids. Cellular senescence was shown to be present in experimental RN. Because fibrosis is the end stage of RN, blocking extracellular matrix deposition may be promising targets for future therapies. Biomarkers to diagnose and assess progression and severity of RN are still missing and need to be identified. They may indicate novel pathways for future research and therapeutic targets in RN.

## Data Availability

Not applicable.

## Abbreviations

AKI: Acute kidney injury
ARN: Acute radiation nephropathy
ACE: Angiotensin-converting enzyme
AT I: Angiotensin I
AT II: Angiotensin II
BED: Biologically effective dose
BMT: Bone marrow transplantation
CKD: Chronic kidney disease
CRN: Chronic radiation nephropathy
DSB: Double-stranded breaks
DAMP: Damage-associated molecular patterns
EETs: Epoxyeicosatrienoic acids
H&E: Hematoxylin and eosin
IMRT: Intensity modulated radiotherapy
MRT: Magnetic resonance tomography
NO: Nitric oxide
QUANTEC: Quantitative Analyses of Normal Tissue Effects in the Clinic
RAAS: Renin–angiotensin–aldosterone system
RN: Radiation nephropathy
RT: Radiotherapy
ROS: Reactive oxygen species
OS: Oxidative stress
TBI: Total body irradiation
TGF-β: Transforming growth factor beta
TMA: Thrombotic microangiopathy
VMAT: Volumetric arc therapy
